# IgG Suppresses Antibody Responses to Sheep Red Blood Cells in Double Knock-Out Mice Lacking Complement Factor C3 and Activating Fcγ-Receptors

**DOI:** 10.3389/fimmu.2020.01404

**Published:** 2020-07-08

**Authors:** Jessica C. Anania, Annika Westin, Birgitta Heyman

**Affiliations:** Department of Medical Biochemistry and Microbiology, Uppsala University, Uppsala, Sweden

**Keywords:** FcgR, complement—immunological term, IgG-mediated immune suppression, Rhesus prophylaxis, antibody feedback

## Abstract

Antigen-specific IgG antibodies, passively administered together with erythrocytes, prevent antibody responses against the erythrocytes. The mechanism behind the suppressive ability of IgG has been the subject of intensive studies, yet there is no consensus as to how it works. An important question is whether the Fc-region of IgG is required. Several laboratories have shown that IgG suppresses equally well in wildtype mice and mice lacking the inhibitory FcγIIB, activating FcγRs (FcγRI, III, and IV), or complement factor C3. These observations consistently suggest that IgG-mediated suppression does not rely on Fc-mediated antibody functions. However, it was recently shown that anti-KEL sera failed to suppress antibody responses to KEL-expressing transgenic mouse erythrocytes in double knock-out mice lacking both activating FcγRs and C3. Yet, in the same study, antibody-mediated suppression worked well in each single knock-out strain. This unexpected observation suggested Fc-dependence of IgG-mediated suppression and prompted us to investigate the issue in the classical experimental model using sheep red blood cells (SRBC) as antigen. SRBC alone or IgG anti-SRBC together with SRBC was administered to wildtype and double knock-out mice lacking C3 and activating FcγRs. IgG efficiently suppressed the IgM and IgG anti-SRBC responses in both mouse strains, thus supporting previous observations that suppression in this model is Fc-independent.

## Introduction

Passively administered IgG can almost completely suppress an antibody response to its specific antigen. The mechanism behind this phenomenon remains elusive, although antibody-mediated immune suppression was first described already in 1909 (1). Despite this, the ability of passively administered IgG to suppress antibody responses to erythrocytes has been used successfully in the clinic. Administration of IgG anti-Rhesus factor D (RhD) to RhD-negative women, at risk of becoming immunized against RhD-positive fetal erythrocytes following transplacental hemorrhage, has proven very efficient in preventing haemolytic disease of the fetus and newborn (2, 3).

Central to understanding the mechanism behind IgG-mediated suppression is to determine whether it requires the Fc-portion of IgG or not. Erythrocyte clearance, complement-mediated lysis or inhibition of B cell activation through co-crosslinking of the negatively regulating FcγRIIB and BCR would all require the IgG Fc-portion. In contrast, masking of epitopes, preventing the erythrocytes from being recognized by BCR, would function without the Fc-portion. Whether trogocytosis, where antibodies induce loss of specific epitopes and thereby lack of induction of an antibody response, is Fc-dependent is unclear since both Fc-dependent (4, 5) and Fc-independent (6, 7) modulation has been observed. The obvious way to determine Fc-dependence is to test whether F(ab′)_2_ fragments can suppress or not. However, such investigations have given discrepant results, some showing that F(ab′)_2_ fragments do suppress (8–10) and others that they do not (11–15).

An alternative approach to determine Fc-dependence has been to test whether IgG suppresses antibody responses in gene targeted mice lacking FcγRs or complement. IgG suppresses efficiently in mice lacking FcγRIIB (FcγRIIB KO) (10, 16–18), activating FcγRs (owing to loss of the common FcR gamma chain, FcRγ KO) (10, 17, 18), both FcγRIIB and activating FcγRs (FcγRIIB × FcRγ double KO) (10), or FcRn (β2-microglobulin KO) (10). Studying suppression of antibody responses in complement-deficient mice is complicated by the fact that lack of complement factors 1, 2, 3, 4, (C1, C2, C3, C4) as well as of complement receptors 1 and 2 (CR1/2) leads to severely impaired antibody responses (19). IgG responses are already extremely low in C-deficient animals immunized with SRBC and a possible suppression caused by passively administered IgG is therefore difficult to assess (18). IgM responses to SRBC are also reduced but still detectable, and are efficiently suppressed by IgG in C1q KO, C3 KO, and CR1/2 KO mice (18). Furthermore, in an experimental system where the antibody response to murine transgenic erythrocytes expressing the entire human KEL glycoprotein (KEL-RBC) was studied, IgG successfully suppressed in C3 or FcRγ KO mice (20). Thus, isolated lack of either FcγRs or complement does not seem to affect the ability of IgG to suppress antibody responses to erythrocytes.

However, using this same KEL-RBC model, no suppression was observed in double KO mice (DKO) lacking both C3 and FcRγ (20). This suggests that complement and FcγRs act redundantly and that suppression is Fc-dependent. This DKO strain is hitherto the only mouse strain found where IgG-mediated suppression does not occur. These findings were surprising in light of the abundance of previous data pointing to Fc-independence and also because IgG responses against xenogeneic erythrocytes in C3-deficient mice are extremely low (18).

The aim of the present study was to determine whether the inability of IgG to suppress in (FcRγ × C3) DKO mice immunized with allogeneic erythrocytes also applies to the response to SRBC, the classical antigen used in studies of suppression. Mice lacking both C3 and FcRγ were bred from the single KO strains and immunized with IgG anti-SRBC and SRBC, or with SRBC alone. IgG consistently suppressed the IgM- as well as the IgG anti-SRBC response both in WT and DKO mice.

## Materials and Methods

### Mice

To obtain (C3 × FcRγ) double KO mice, C3 KO (Jackson Laboratories, Bar Harbor, ME, USA) and FcRγ KO mice [a gift from Dr. J. V. Ravetch (21) backcrossed in-house for 10 generations to BALB/c from Bommice, Ry, Denmark] were crossed, and the resulting F1 mice intercrossed. The F2 mice were typed for FcRγ alleles in a PCR using the primers: Neo (5′-CTC GTG CTT TAC GGT ATC GCC-3′), OL4087 (5′-ACC CTA CTC TAC TGT CGA CTC AAG-3′), and OL4081 (5′-CTC ACG GCT GGC TAT AGC TGC CTT-3′) yielding 224 (WT) and 302 (FcRγ KO) bp bands. F2 mice homozygous for the mutant FcRγ allele were bled and C3 levels in sera were assayed by radial immunodiffusion using a goat anti-mouse C3 antiserum (CooperBiomedical Inc., Malvern, PA). Offspring from homozygous FcRγ KO mice with undetectable C3 titers (<1:2; no detectable precipitation line) were used in experiments as double KO mice (WT mice had C3 titers ≥1:32). The lack of C3 was confirmed in a PCR using the primers 5′-ATC TTG AGT GCA CCA AGC C-3′ and 5′-GGT TGC AGC AGT CTA TGA AGG-3′ (WT) and 5′-CTT GGG TGG AGA GGC TAT TC-3′ and 5′-AGG TGA GAT GAC AGG AGA TC-3′ (C3 KO) yielding 350 (WT) and 280 (C3 KO) bp bands (22). The founding C3 KO mice were on C57BL/6 and FcRγ KO mice on BALB/c background. As WT controls we therefore used (C57BL/6 × BALB/c) F1 mice, resulting from mating BALB/c from Bommice, Ry, Denmark and C57BL/6JBomTac (C57BL/6) mice from Taconic, Ejby, Denmark.

Mice within each experiment were matched for age and sex. Animals were bred and maintained in the animal facilities at the National Veterinary Institute (Uppsala, Sweden). This study was carried out in accordance with the recommendations of the Uppsala Animal Research Ethics Committee, and the protocol was approved by the Uppsala Animal Research Ethics Committee (permit numbers C25/15 and Dnr 5.8.18-02583/2018).

### Antigen and IgG Anti-SRBC Used for Immunization

SRBC were obtained from Håtunalab AB (Håtunaholm, Sweden) and were stored in sterile Alsever's solution at 4°C. IgG anti-SRBC of the Ig^a^ and Ig^b^ allotypes were prepared as described (23). Briefly, BALB/c and C57BL/6 mice were immunized i.v. with 10% SRBC three times. Sera from blood obtained 4 weeks after the last immunization were run over a Protein-A Sepharose column (Amersham Pharmacia Biotech, Uppsala, Sweden). The eluted IgG fractions were dialyzed, concentrated and stored at −20° until use.

### Immunization and Blood Sampling

Mice were immunized with the indicated amounts and allotypes of IgG anti-SRBC in 100 μl PBS followed within 1 h by SRBC in 100 μl PBS. Controls received IgG anti-SRBC or SRBC alone. All immunizations were done in one of the lateral tail veins. To analyze IgG-responses, mice were bled from the ventral tail artery into individual tubes and the blood allowed to clot at 4°C overnight. Sera were then removed, spun at 13,000 rpm, cell-free fractions collected and frozen at −20°C until use in ELISA.

### Plaque Forming Cell Assay

To analyze IgM-responses, a modified version of the Jerne haemolytic plaque assay (24) was used and details have been described previously (25). Briefly, spleens were removed 5 days after immunization and appropriately diluted single cell suspensions were mixed with agarose (at 45°C), SRBC, and complement (guinea pig serum). The mix was immediately spread on a microscope slide and incubated for 3 h at 37°C in a humid chamber. Haemolytic plaques, each representing a single spleen cell producing IgM anti-SRBC, were counted “blindly.” All dilutions were made in Hank's balanced salt solution, HBSS. Duplicate samples were analyzed. In the event of no detectable plaque forming cells (PFC) in 1:100 of a spleen, the spleen was assigned 50 PFC in order to allow further calculations.

### ELISA

The ELISA used to detect IgG anti-SRBC has been described previously (25). Briefly, ELISA plates were coated with SRBC and incubated with appropriately diluted sera from immunized mice. To allow distinction between the passively administered IgG (allotype IgG^a^) and the IgG produced endogenously as a response to the SRBC-immunization, an allotype-specific ELISA, not recognizing IgG of the a allotype, was used. Antibody responses were assayed with an appropriately diluted 1:1 mix of biotinylated monoclonal anti-IgG1^b^ (clone B68-2) and anti-IgG2a^b^ (clone 5.7). To test the allotype specificity of the ELISA, also biotinylated monoclonal anti-IgG1^a^ (clone 10.9) and anti-IgG2a^a^ (clone 8.3) were used. All allotype specific antibodies were from BD Pharmingen. To test the allotype specificity of the ELISA, also biotinylated monoclonal IgG1^a^ and IgG2a^a^ were used. The biotinylated mAbs were added and plates incubated at 4°C overnight. After washing, alkaline phosphatase-conjugated streptavidin (BD Pharmingen, San Jose, CA, USA) was added for 3 h at room temperature. Plates were developed using the substrate p-nitrophenylphosphate (Sigma-Aldrich). Absorbance at 405 nm was measured after 60 min incubation with substrate. As positive and negative controls for the allotype specificity of the ELISA, sera from SRBC-immunized BALB/c (IgG^a^) and C57BL/6 (IgG^b^) were tested in a crisscross fashion: in the anti-a allotype ELISA only BALB/c sera were positive and vice versa. That the ELISA does not detect the passively administered IgG anti-SRBC antibodies is also evidenced by the lack of a detectable response in mice transferred with IgG alone.

### Statistical Analysis

Statistical differences between the groups were determined by two-way ANOVA. *P* < 0.001 is symbolized by ^***^p < 0.01 by ^**^p < 0.05 by ^*^p > 0.5 by ns.

### Ethics Approval

This study was carried out in accordance with the recommendations of the Uppsala Animal Research Ethics Committee, and the protocol was approved by the Uppsala Animal Research Ethics Committee.

## Results

### IgG Suppresses IgM Anti-SRBC Responses in (FcRγ × C3) DKO Mice

**Figure 1:**
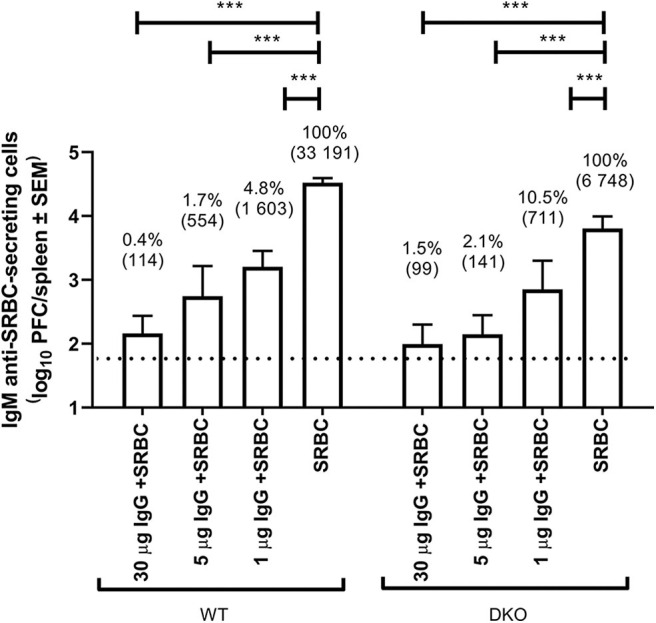
IgG suppresses IgM anti-SRBC responses in (FcRγ × C3) DKO mice. WT or (FcRγ × C3) DKO mice (*n* = 3–7/group) were immunized i.v. with 5 × 10^7^ SRBC alone or with 30, 5, or 1 μg polyclonal IgG^a^ anti-SRBC followed within 1 h by 5 × 10^7^ SRBC. Negative controls received 30, 5, or 1 μg IgG^a^ anti-SRBC alone (*n* = 2–4/group). Spleens were harvested 5 days later and the number of spleen cells producing IgM anti-SRBC were determined in a direct PFC assay. The dotted line represents the average number of PFC in mice immunized with IgG alone. In addition to log_10_ PFC/spleen (y-axis), values are also shown as geometrical mean (within parenthesis) and as percent of the response in control mice immunized with SRBC alone. Statistical differences between the groups were determined by two-way ANOVA. ***p < 0.001. This experiment was performed an additional 4 times using 50 or 30 μg of IgG (see Table 1). Not shown in the figure are the statistical differences between WT and DKO given the same treatment: 30 μg IgG + SRBC –> WT vs. 30 μg IgG + SRBC –> DKO (ns, not significant); 5 μg IgG + SRBC –> WT vs. 5 μg IgG + SRBC –> DKO (ns); 1 μg IgG + SRBC –> WT vs. 1 μg IgG + SRBC –> DKO (ns); SRBC –> WT vs. SRBC –> DKO (*p* < 0.001).

WT and (FcRγ × C3) DKO mice were immunized with IgG anti-SRBC and SRBC, SRBC alone, or IgG anti-SRBC alone and the number of IgM anti-SRBC-producing spleen cells (measured as PFC) were assayed 5 days after immunization (Figure 1). As expected, the number of PFC were lower in DKO mice (6 748/spleen) than in WT mice (33 191/spleen) immunized with SRBC. However, in both strains all doses (1–30 μg) of the passively administered IgG significantly inhibited the PFC response. For example, 30 μg of IgG co-administered with SRBC left only 0.4% of the control response in WT and 1.5% in DKO mice, thus resulting in 99.6 and 98.5% suppression, respectively.

Similar experiments were performed an additional four times independently and are all shown in Table 1. They consistently resulted in efficient suppression in both WT and DKO mice (Table 1). Fifty (Exp. 1) or 30 μg (Exp. 2–3) of SRBC-specific IgG^a^ resulted in <1% of the control response in WT mice and <4.5% in DKO mice. In comparison, 30 μg of SRBC-specific IgG^b^ left 0.9% of the response in WT and 1.5% in DKO mice (Exp. 4). This slightly lower relative suppression observed in DKO mice can be explained by their overall lower response to SRBC alone, caused by lack of C3. For example, in Exp. 1 (Table 1) IgG induced complete suppression resulting in no detectable PFC (assigned 50 PFC/spleen) in either strain. However, because WT mice had a control response of 8 331 PFC/spleen and DKO mice of 1 103 PFC/spleen, the relative suppression in WT mice was 0.6% and in DKO mice 4.5%. This illustrates that although the antibody responses were maximally suppressed in both strains, the relative suppression was smaller in DKO mice because the methodology only allows a minimum of 50 PFC/spleen. In summary, passively transferred IgG consistently and efficiently suppressed the IgM anti-SRBC responses both in WT and DKO mice.

**Table 1:** IgG suppresses IgM anti-SRBC responses both in WT and (C3 × FcγR) DKO mice.

Exp^*^	**Immunization^*†*^**	**WT**		**DKO**	
		**Log_**10**_ PFC/spleen (geometric mean) Significance**	**%^§^**	**Log_**10**_ PFC/spleen (geometric mean) Significance**	**%^§^**
1	5 × 10^7^ SRBC	3.92 ± 0.05 (8 331)	100	3.04 ± 0.43 (1 103)	100
	5 × 10^7^ SRBC + 50μg IgG^a^	1.70 ± 0.00 (50)	0.6	1.70 ± 0.00 (50)	4.5
		^***^		^***^	
2	5 × 10^7^ SRBC	4.67 ± 0.14 (46 ° 609)	100	3.79 ± 0.37 (6 197)	100
	5 × 10^7^ SRBC + 30μg IgG^a^	2.36 ± 0.24 (228)	0.5	2.28 ± 0.35 (189)	3.1
		^***^		^***^	
3	5 × 10^7^ SRBC	4.59 ± 0.04 (38 600)	100	4.06 ± 0.15 (11 532)	100
	5 × 10^7^ SRBC + 30μg IgG^a^	2.17 ± 0.30 (149)	0.4	2.22 ± 0.28 (165)	1.4
		^***^		^***^	
4	5 × 10^7^ SRBC	4.72 ± 0.06 (52 902)	100	4.10 ± 0.24 (12 498)	100
	5 × 10^7^ SRBC + 30μg IgG^b^	2.66 ± 0.57 (458)	0.9	2.28 ± 0.97 (189)	1.5
		^***^		^***^	

* 1–4 represents four independent experiments with n = 3–8 in each group.

† *Mice were immunized with IgG anti-SRBC alone, SRBC alone, or IgG anti-SRBC + SRBC. Geometric mean of the PFC numbers/spleen in groups immunized with IgG alone ranged from 50 to 324 (not included in table)*.

Statistical significance calculated by 2-way ANOVA;

*** *p < 0.001*.

§ *Percentage of the control responses (number of PFC in mice immunized with SRBC alone, 100%) that remains in mice immunized with IgG anti-SRBC + SRBC*.

### IgG Suppresses IgG Anti-SRBC Responses in (FcRγ × C3) DKO Mice

**Figure 2:**
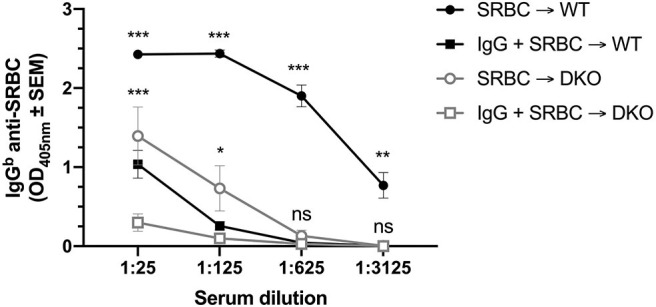
IgG suppresses IgG anti-SRBC responses in (FcRγ × C3) DKO mice. WT or (FcRγ × C3) DKO mice were immunized i.v. with 5 × 10^7^ SRBC alone, 30 μg polyclonal IgG^a^ anti-SRBC alone, or with 30 μg polyclonal IgG^a^ anti-SRBC + 5 × 10^7^ SRBC (*n* = 9 or 10 mice/group). Sera obtained 20 days after immunization were tested in ELISA. IgG anti-SRBC levels in WT or DKO mice immunized with IgG alone were similar to levels in the blanks (not shown). Statistical differences between the groups were determined by two-way ANOVA. Statistical differences between WT mice immunized with SRBC or IgG + SRBC are shown above the upper curve (black) and statistical differences between DKO mice immunized with SRBC or IgG + SRBC above the middle curve (gray). ***p < 0.001; **p < 0.01; *p < 0.05. The data shown are pooled from two separate experiments. The experiment was performed an additional time, using a 10-fold lower dose (5 × 10^6^ SRBC/mouse) and 30 μg IgG^a^ anti-SRBC: WT mice were suppressed down to a serum dilution of 1:625 and DKO mice suppressed at a serum dilution of 1:25.

To address whether IgG could also suppress IgG anti-SRBC responses, WT and (FcRγ × C3) DKO mice were immunized with polyclonal IgG^a^ anti-SRBC and SRBC, SRBC alone, or polyclonal IgG^a^ anti-SRBC alone. Three weeks later, the mice were bled and their sera analyzed by anti-SRBC ELISA (Figure 2). Single C3 KO mice have very low IgG-responses after immunization with SRBC (18) and a similar result was observed in the DKO mice. Nevertheless, IgG significantly suppressed the antibody responses both in WT and DKO mice when sera diluted 1:25 or 1:125 were analyzed in two independent experiments (Figure 2). At higher dilutions (1:625-1:3125), the IgG anti-SRBC levels in DKO control mice, immunized with SRBC alone, were very low and no significant suppression could be detected. In contrast, high levels of IgG anti-SRBC was observed in WT control mice and IgG-mediated suppression was significant also when the highest serum dilution (1:3125) was analyzed. In a third experiment, using a 10-fold lower dose of SRBC, suppression in WT mice was observed down to a serum dilution of 1:625 while suppression in DKO mice could only be detected at the 1:25 serum dilution.

In summary, similar to C3 KO mice, (FcRγ × C3) DKO mice have severely impaired IgG-responses after immunization with SRBC. Nevertheless, IgG consistently suppressed the IgG anti-SRBC responses in the DKO as well as in WT mice.

## Discussion

Many hypotheses have been proposed to explain how IgG antibodies are able to nearly completely suppress antibody responses. They can be divided into those that require the Fc-region of IgG and those that do not. An interesting, but yet unresolved, question is how important glycosylation of the Fc-regions is. Poor glycosylation results in poor FcR binding (26) and it has been reported that the anti-inflammatory activity of IgG is reduced when the Fc-region is not sialylated (27). Involvement of immunomodulatory cytokines such as TGFβ has also been discussed, in particular in anti-RhD prophylaxis (28). One of the most commonly proposed ideas has been that BCR and FcγRIIB are co-crosslinked by IgG-antigen complexes, thus allowing negative signaling via the ITIM motif in FcγRIIB to negatively regulate antigen specific B cells. However, it is unlikely that this plays a major role because IgG-mediated suppression is unperturbed in mice lacking this receptor (10, 16–18). Enhanced clearance, induced by the passively administered IgG, has also been frequently discussed. In models using SRBC, this is not likely to play a major role because these erythrocytes are eliminated within minutes whether IgG is co-administered or not (29). Moreover, IgG administered several days after SRBC, i. e. when no antigen remains in the circulation, induces suppression (13, 16). Also in allogeneic experimental models, it is unlikely that clearance is the single explanation as monoclonal antibodies, which did not increase clearance, nevertheless suppressed the antibody response (30). Complement, activated by the passively administered IgG bound to the erythrocyte surface, could lead to either increased phagocytosis or lysis of erythrocytes, thus rendering them less immunogenic. However, suppression works well in the absence of complement (18, 20) and monoclonal IgG antibodies that do not activate complement can suppress (31). These observations argue against an exclusive role for complement in IgG-mediated suppression.

The aim of the current study was to elucidate whether simultaneous loss of activating FcγRs and complement activity led to reduced ability of IgG to suppress antibody responses, as shown to be the case in an experimental system using allogeneic erythrocytes (20). We here investigated whether SRBC-specific IgG, passively administered together with SRBC, can suppress the antibody response in DKO mice lacking both C3 and activating FcγRs. Efficient suppression of the IgM anti-SRBC response, ranging from 95.5 to 98.6%, was consistently observed in DKO mice when 30 or 50 μg IgG was administered and as little as 1 μg IgG caused 89.5% suppression (Table 1, Figure 1). Significant suppression of the serum IgG response was also observed both in WT and DKO mice (Figure 2). The relative magnitude of suppression in the two strains is difficult to compare owing to the enormous variation in responses to SRBC alone between them.

In a previous report, anti-KEL sera from mice immunized with murine transgenic KEL-RBC did not suppress the IgG anti-KEL response in (FcRγ × C3) DKO mice although suppression worked well in each single KO strain (20). The reason for the difference between the findings of Liu et al. and the present study is not understood. However, it should be noted that the experimental systems used are quite different. Allogeneic mouse erythrocytes, expressing the transgenic glycoprotein KEL, were used as antigen by Liu et al., while the present study used xenogeneic SRBC. As suppressive agent, unfractioned whole serum from mice pre-treated three times with 100 μg poly (I:C) and then immunized three times with KEL-RBC was used by Liu et al. In comparison, we used IgG obtained after affinity chromatography on Protein A-Sepharose of sera from mice hyperimmunized with SRBC in PBS. Liu et al. used a flow cytometric crossmatch assay, while our read-out was single cells producing SRBC-specific IgM or a traditional ELISA measuring SRBC-specific IgG. Yet another unexplained difference is that DKO mice produced similar levels of anti-KEL IgG as WT mice after immunization with KEL-RBC (20) while our DKO mice were very poor producers of IgG after immunization with SRBC (Figure 2). Antibody responses to SRBC and many other antigens are known to be severely impaired in the absence of early classical complement components, C3, or CR1/2 (19). Generally, IgG responses seem to be more affected than IgM responses (18, 32–34) and this was true also in the present study.

Trogocytosis/antigen modulation has recently been suggested to explain suppression of antibody responses to transgenic epitopes on the erythrocyte membrane, which are recognized by passively transferred epitope-specific IgG or serum (20, 35–38). It is hypothesized that the transferred antibodies cause removal of the epitope to which they bind, and sometimes also of other nearby epitopes on the cell membrane. This would result in lack of an antibody response because the epitopes have been removed. The details on how trogocytosis operates are scarce and both Fc-dependent and Fc-independent mechanisms have been described (4–7). We find it unlikely that this mechanism can explain the complete suppression of antibody responses to all epitopes on SRBC. This would require that trogocytosis destroys all erythrocytes and in experimental systems using transferred allogeneic erythrocytes these remain intact in the circulation for hours or days during conditions where trogocytosis has removed specific epitopes (20, 37).

The efficient suppression observed in our DKO mice reinforces that suppression of SRBC-responses is independent of the IgG(Fc) region. This has implications when considering the mechanism behind IgG-mediated immune suppression. The observation is difficult to reconcile with complement-mediated lysis or increased erythrocyte clearance. Moreover, the successful suppression in FcγRIIB KO mice, as demonstrated in other studies, excludes a single role of this receptor (10, 16–18). In our view, epitope masking remains the most likely explanation for IgG-mediated suppression of antibody responses to SRBC. Direct evidence for this mechanism is difficult to obtain because it would require *in vivo* studies of whether the transfused IgG antibodies prevent interaction between naïve SRBC-specific B cells and the IgG-covered SRBC. Binding studies could be performed *in vitro*, but would rely on suppressive IgG blocking binding to erythrocytes of other antibodies, and not of naïve B cells, which is quite a different situation. Although direct experimental evidence is hard to obtain, epitope masking is compatible with many observations, such as the unperturbed suppression in mice lacking C and/or FcγRs discussed above. Similarly, lack of suppression of T helper cell responses (10, 29, 39), epitope specificity of suppression [except during conditions of high epitope density (25)], suppression by F(ab')_2_ (8–10), IgE (10, 40), and IgM (14), the additive effect of several mAbs (41–43), and suppression by IgG administered several days after SRBC (13, 16, 44) also support epitope masking.

Other observations argue against epitope masking as the explanation for IgG-mediated suppression. Although F(ab')_2_ fragments, as mentioned above, have indeed been reported to suppress (8–10) other workers find that they cannot suppress (11–15). Hypothetically, the conflicting observations may be explained by increased elimination of F(ab')_2_ fragments owing to loss of binding to FcRn, which normally protects IgG from proteolysis (45), but this remains to be elucidated. Moreover, in studies using murine allogeneic erythrocytes, suppression was shown to occur in the apparent absence of epitope masking (30, 36). Obviously, the lack of suppression against allogeneic KEL-RBC in DKO mice (20) also points to other mechanisms. At present, it cannot be excluded that suppression of antibody responses to allogeneic erythrocytes takes place through different mechanisms than suppression against xenogeneic erythrocytes.

In conclusion, we find that the data presented herein, which clearly demonstrates that IgG can suppress without involvement of either complement or activating FcγRs, strengthens epitope masking as an important mechanism behind IgG-mediated suppression of antibody responses to xenogeneic erythrocytes.

## Data Availability Statement

The datasets generated for this study are available on request to the corresponding author.

## Ethics Statement

The animal study was reviewed and approved by Uppsala Animal Research Ethics Committee.

## Author Contributions

JA, AW, and BH designed the experiments and wrote the manuscript. JA and AW performed the experiments. All authors contributed to the article and approved the submitted version.

## Conflict of Interest

The authors declare that the research was conducted in the absence of any commercial or financial relationships that could be construed as a potential conflict of interest.
